# Picket-Fence Technique in Surgical Treatment of Cerebral Aneurysms and Role of Intraoperative Videoangiography in Aneurysm Surgery

**DOI:** 10.3390/medicina61111974

**Published:** 2025-11-04

**Authors:** Utku Özgen, Mehmet Osman Akçakaya, Talat Kırış

**Affiliations:** 1 Department of Neurosurgery, American Hospital, 34365 Istanbul, Türkiye; talatkrs@gmail.com; 2 Department of Neurosurgery, Florence Nightingale Hospital, 34381 Istanbul, Türkiye; moakcakaya@gmail.com

**Keywords:** picket-fence technique, giant aneurysm, videoangiography

## Abstract

*Background and Objectives*: To evaluate factors affecting aneurysm rupture, present our surgical experience with intracranial aneurysms, specifically using the picket-fence clipping technique for giant aneurysms, and highlight the complementary roles of sodium fluorescein (Na-Fl) and indocyanine green (ICG) videoangiography in enhancing surgical precision and patient outcomes. *Materials and Methods*: We retrospectively analyzed 47 patients who underwent microsurgical clipping of intracranial aneurysms with intraoperative Na-Fl and ICG videoangiography between September 2015 and February 2024. We assessed relationships between patient comorbidities, family history of subarachnoid hemorrhage (SAH), smoking history, aneurysm location and size, and SAH occurrence. Concordance between intraoperative videoangiography and postoperative digital subtraction angiography (DSA) for detecting residual aneurysms was also evaluated. *Results*: Of the 47 patients (31 female, 16 male; mean age 51.78 ± 11.16 years), 11 (23.4%) presented with SAH. The most common aneurysm location was the middle cerebral artery (MCA) (68.1%). Hypertension and smoking history were significantly higher in the hemorrhage group (*p* < 0.05). Aneurysm size and anterior communicating artery (AComA) location were also significantly associated with hemorrhage (*p* < 0.05). Aneurysm size demonstrated significant discriminative power for hemorrhage [AUC: 0.884 (0.827–0.941)], with a cutoff of 7.1 mm yielding 90.9% sensitivity and 94.4% specificity. Five giant MCA aneurysms were treated with the picket-fence technique, with intraoperative ICG and Na-Fl confirming parent artery patency and complete aneurysm occlusion, subsequently confirmed by postoperative DSA. Small remnants were detected in 2 cases (4.26%) on postoperative DSA, both in distal ACA aneurysms, which were also detected by intraoperative videoangiography. *Conclusions*: Hypertension, smoking history, aneurysm size, and location were important predictors of aneurysm rupture. Intraoperative ICG and Na-Fl videoangiography provide real-time, high-resolution visualization crucial for complex intracranial aneurysm surgery, including the picket-fence technique for giant aneurysms. Their complementary use enhances surgical safety, guides intraoperative decision-making, and contributes to improved outcomes in challenging cases.

## 1. Introduction

The treatment of intracranial aneurysms has undergone major advancements in recent decades, with both microsurgical clipping and endovascular techniques such as coiling and flow diversion widely used in clinical practice. Endovascular treatment is often favored for its minimally invasive nature, reduced hospital stay, and lower perioperative morbidity, especially in elderly or high-risk patients [1,2]. However, surgical clipping remains a highly effective and durable treatment, particularly in younger patients and aneurysms with wide necks, complex morphology, or location-related challenges [3]. In the case of giant aneurysms (>25 mm), endovascular options may be limited due to higher recurrence rates, incomplete occlusion, and thromboembolic complications [4]. The morbidity and mortality rates following rupture of giant aneurysms are substantially higher than those of smaller aneurysms, with mortality rates reported between 20 and 30% [5].

Given the complex anatomy, wide necks, incorporation of major vessels, and presence of thrombus or calcification, the surgical management of giant aneurysms poses significant technical challenges. In selected cases, microsurgical clipping remains a critical treatment modality, especially when endovascular approaches are contraindicated or have failed. One advanced microsurgical technique employed in these scenarios is the picket-fence technique, which involves the placement of multiple tandem clips in an overlapping, staggered fashion along the aneurysm neck. This method allows for controlled closure of large or calcified necks, improves clip stability, and enhances the ability to preserve adjacent vessels while achieving complete aneurysm obliteration.

Intraoperative visualization tools have become essential in guiding such complex procedures. Indocyanine green (ICG) videoangiography, performed under near-infrared illumination, provides real-time assessment of vascular patency and clip efficacy, enabling immediate intraoperative corrections [6]. Sodium fluorescein (Na-Fl), used in conjunction with a yellow 560 nm filter on the operating microscope, offers an additional method for intraoperative vascular imaging. It allows dynamic visualization of blood flow and may be particularly beneficial in situations where ICG is limited, such as in small perforators or slow flow states [7].

This study aims to evaluate the factors affecting aneurysm rupture, present our surgical experience in the treatment of intracranial aneurysms as well as using the picket-fence clipping technique in giant aneurysms, and also highlight the complementary roles of sodium fluorescein and ICG videoangiography in enhancing surgical precision, intraoperative decision-making, and ultimately, patient outcomes.

## 2. Materials and Methods

A total of 47 patients who underwent microsurgical clipping of intracranial aneurysms with the assistance of Na-Fl and ICG videoangiography between September 2015 and February 2024 were retrospectively evaluated in this study. All procedures were performed by a single neurosurgeon at two centers. This study is carried out in accordance with the World Medical Association Declaration of Helsinki. Privacy rights of human subjects have been observed, and informed consent was obtained from all patients.

Patients with ruptured/unruptured aneurysms were included in the current study. The relationships between patients’ comorbidities, family history of subarachnoid hemorrhage, history of smoking, aneurysm location, and aneurysm size with subarachnoid hemorrhage were evaluated, as well as the concordance between intraoperative videoangiography and postoperative DSA in detecting residual aneurysms (Table 1).

Patients were evaluated with preoperative cranial computed tomography (CT) scans and CT angiography. DSA was performed in all cases. All patients underwent surgery under general anesthesia. Surgical microscopes equipped with YELLOW 560 nm and INFRARED 800 nm filters were used in all patients. (PENTERO 900/KINEVO 900, Carl Zeiss, Meditec, Oberkochen, Germany). Concordance between intraoperative videoangiography and postoperative DSA for detecting residual aneurysms was also evaluated.

All patients underwent standard microsurgical procedures by performing pterional craniotomy for clipping. Following the exposure of the aneurysm, a videoangiography with Na-Fl was performed. After exposing the aneurysm through a craniotomy of appropriate size, 1 mL (100 mg) of Na-Fl was intravenously injected in all patients for videoangiography. Na-Fl angiography provides an opportunity for the surgeon for real-time manipulation of the vessels. Following Na-Fl videoangiography, 25 mg of ICG was intravenously administered to all patients, and ICG videoangiography was performed. If necessary, an additional 1 mL (100 mg) of 10% Na-Fl and 25 mg of ICG were administered for repeated videoangiography assessments. Vessel fluorescence was visible within seconds and was cleared within 10 min, allowing for additional injections. The total dosage did not exceed 5 mL (500 mg) of Na-Fl and 5 mg/kg of ICG.

### 2.1. Picket-Fence Techique in Giant Aneurysms

Sugita and his colleagues advanced the use of fenestrated clipping by developing a diverse set of clips with varying blade lengths, angles, and fenestration diameters [8,9]. He later introduced the concept of tandem clipping as an innovative technique. In contrast to the tandem fenestrated clipping method, Yang described a different approach in the literature known as the “fenestration tube” technique, in which fenestrated clips are sequentially stacked to form tubular structures that help reconstruct efferent branch arteries [4]. The picket fence clipping technique is a method used in giant and complex aneurysms where direct clipping of the aneurysm neck is not feasible due to factors such as atherosclerosis, complex anatomy, calcification, or difficulty in preserving parent arteries. In this technique, the aneurysm is occluded using sequentially placed fenestrated or straight clips. This method involves the sequential application of multiple fenestrated or straight clips, aligned in a row, mimicking the structure of a “picket fence.” The technique allows for progressive occlusion of the aneurysmal sac while maintaining optimal control over adjacent perforating and parent arteries. It also facilitates customized clip positioning in cases where the aneurysm neck is irregular or inaccessible from a single clip trajectory.

### 2.2. Illustrative Case-I

**Figure 1:**
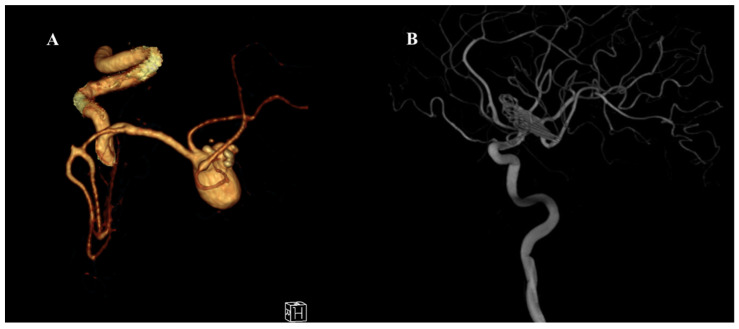
Preoperative and postoperative angiographic images of a giant middle cerebral artery (MCA) aneurysm. (**A**) Three-dimensional CT angiography reconstruction demonstrating a giant MCA bifurcation aneurysm with multilobulated morphology and complex neck anatomy. (**B**) Postoperative digital subtraction angiography (DSA) image showing complete occlusion of the aneurysm following surgical clipping using the picket-fence technique. The parent vessels and MCA branches remain patent without evidence of residual aneurysm or stenosis.

A 43-year-old male patient with no known comorbidities and an intact neurological examination was incidentally found to have a calcified giant bifurcation aneurysm of the left MCA (Figure 1).

**Figure 2:**
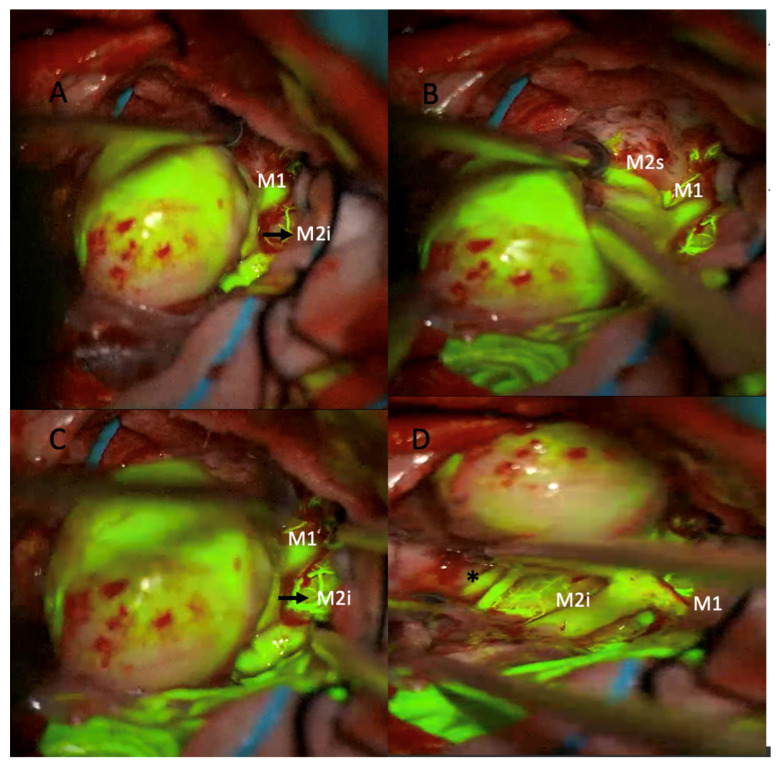
Intraoperative microscopic views of a giant middle cerebral artery (MCA) aneurysm during microsurgical clipping using sodium fluorescein videoangiography under a yellow 560 nm filter. (**A**) The large aneurysmal sac is seen adjacent to the M1 segment and the inferior division of the M2 segment (M2i). (**B**) After further dissection, the superior division of the M2 segment (M2s) is identified. Fluorescein fluorescence highlights the patent parent and branch vessels. (**C**) Confirmation of perfusion in the M1 and M2i segments is achieved prior to clip application. (**D**) Post-clipping image showing preserved fluorescence in the M1 and M2i segments, indicating intact flow (* asterisk marks the patent M2 inferior trunk branches).

**Figure 3:**
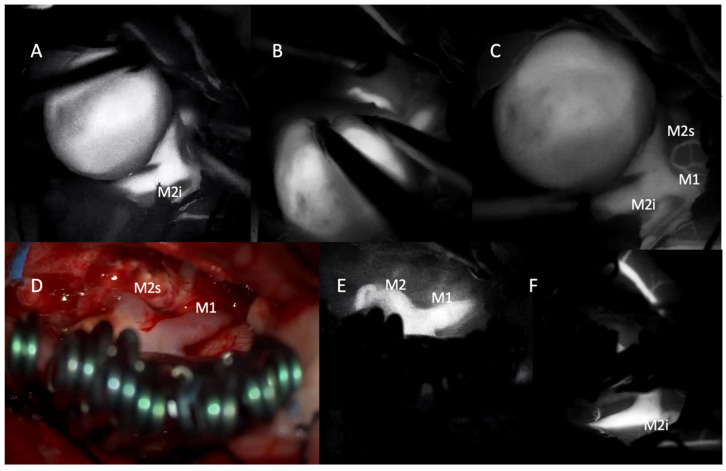
Intraoperative indocyanine green (ICG) videoangiography images demonstrating the microsurgical management of a giant middle cerebral artery (MCA) aneurysm. (**A**–**C**) Pre-clipping ICG videoangiography showing the large aneurysmal sac and its relationship to the M1 segment and the inferior (M2i) and superior (M2s) divisions of the M2 segment. Vessel filling indicates patent flow in adjacent branches. (**D**) Intraoperative photograph following clip placement using the picket-fence technique with multiple fenestrated clips. The M1 and M2 segments are preserved, and the aneurysm is excluded from the circulation. (**E**,**F**) Post-clipping ICG videoangiography confirms uninterrupted flow in the M1 and both M2 branches (M2s and M2i) with no evidence of residual filling within the aneurysmal sac.

The patient underwent surgery using the picket-fence clipping technique, during which seven Sugita fenestrated clips were applied. Intraoperative sodium fluorescein and ICG videoangiography confirmed complete aneurysm occlusion and preservation of the parent arteries (Figure 2 and Figure 3).

No abnormalities were observed in the patient’s postoperative neurological examination, and postoperative neuroradiological images showed no residual filling of the aneurysm.

### 2.3. Illustrative Case-II

**Figure 4:**
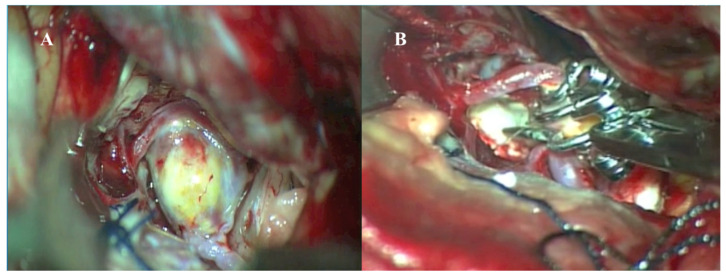
Intraoperative microscopic images of microsurgical clipping of a giant anterior communicating artery (AcomA) aneurysm. (**A**) Intraoperative view showing the exposed, partially thrombosed giant AcomA aneurysm following careful dissection of surrounding neurovascular structures. (**B**) Clipping of the aneurysm using multiple fenestrated clips in a picket-fence configuration to reconstruct the aneurysm neck and preserve flow through the bilateral A1 and A2 segments.

A 55-year-old female patient presented to the emergency department with a sudden onset of severe headache. Diagnostic evaluations revealed a World Federation of Neurosurgical Societies (WFNS) Grade I subarachnoid hemorrhage and an AcomA aneurysm. The patient underwent surgery via a left pterional craniotomy, during which the aneurysm was successfully occluded using the picket-fence technique with four Sugita fenestrated clips (Figure 4).

**Figure 5:**
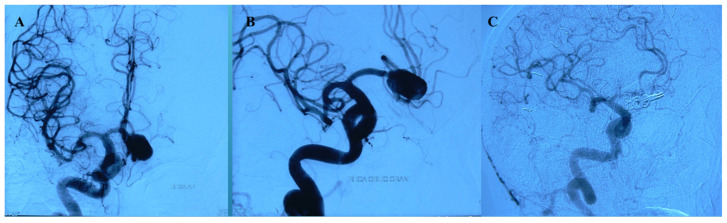
(**A**,**B**) Preoperative DSA showing AcomA aneurysm. (**C**) Postoperative DSA showing multiple clips in a picket-fence configuration.

The procedure was completed without complications. Postoperative DSA showed no residual aneurysmal filling (Figure 5). Her postoperative course was uneventful, and she was discharged in a stable condition.

### 2.4. Statistical Analysis

Mean, standard deviation, median, minimum, maximum value frequency, and percentage were used for descriptive statistics. The distribution of variables is measured by Kolmogorov–Smirnov and Shapiro–Wilk tests. Mann–Whitney U tests were used in the analysis of independent quantitative data with non-normal distribution. The chi-square test was used for the comparison of the qualitative data. The effect level was tested with an ROC curve. The effect level was tested with logistic regression. The kappa test was used in the goodness-of-fit analysis. SPSS 28.0 was used for statistical analyses.

## 3. Results

There were 31 female patients (66.0%) and 16 male patients (34.0%), with a mean age of 51.78 ± 11.16 years (range, 13–80). A total of 47 aneurysms were surgically treated. Eleven aneurysms (23.4%) were presented with SAH at presentation. WFNS grades of the aneurysms with SAH were as follows: Grade I in one patient (8.3%), Grade II in two patients (16.7%), Grade III in four patients (33.3%), and Grade IV in four patients (33.3%). The remaining 36 aneurysms (76.6%) were detected incidentally during neuroimaging performed for unrelated clinical indications. The most common aneurysm location was the MCA, accounting for 32 cases (68.1%). The AComA was the site of 11 aneurysms (23.4%), followed by the anterior cerebral artery (ACA) with three cases (6.4%) and the posterior communicating artery (PComA) with one case (2.1%). Out of 32 MCA aneurysms, five cases (15.6%) were treated using the picket-fence clipping technique. Intraoperative ICG and Na-Fl videoangiography confirmed that the parent arteries remained patent. The technical quality of videoangiography was optimal for all of the aneurysms, and the concordance of intraoperative videoangiography with postoperative DSA is 100%. Postoperative DSA examinations of five patients with MCA aneurysms who were operated on using the picket-fence technique revealed no remnants, and the perforating arteries were observed to be patent.

There was no significant difference between the hemorrhage and non-hemorrhage groups in terms of age, gender distribution, history of subarachnoid hemorrhage, diabetes rate, or the rate of aneurysms in the MCA, PcomA, and ACA (*p* > 0.05) (Table 2). However, the rate of hypertension and history of smoking were significantly higher in the hemorrhage group compared to the non-hemorrhage group (*p* < 0.05) (Table 2).

Additionally, the rate of aneurysms in the AcomA and the aneurysm size were significantly higher in the hemorrhage group than in the non-hemorrhage group (*p* < 0.05) (Table 2).

**Figure 6:**
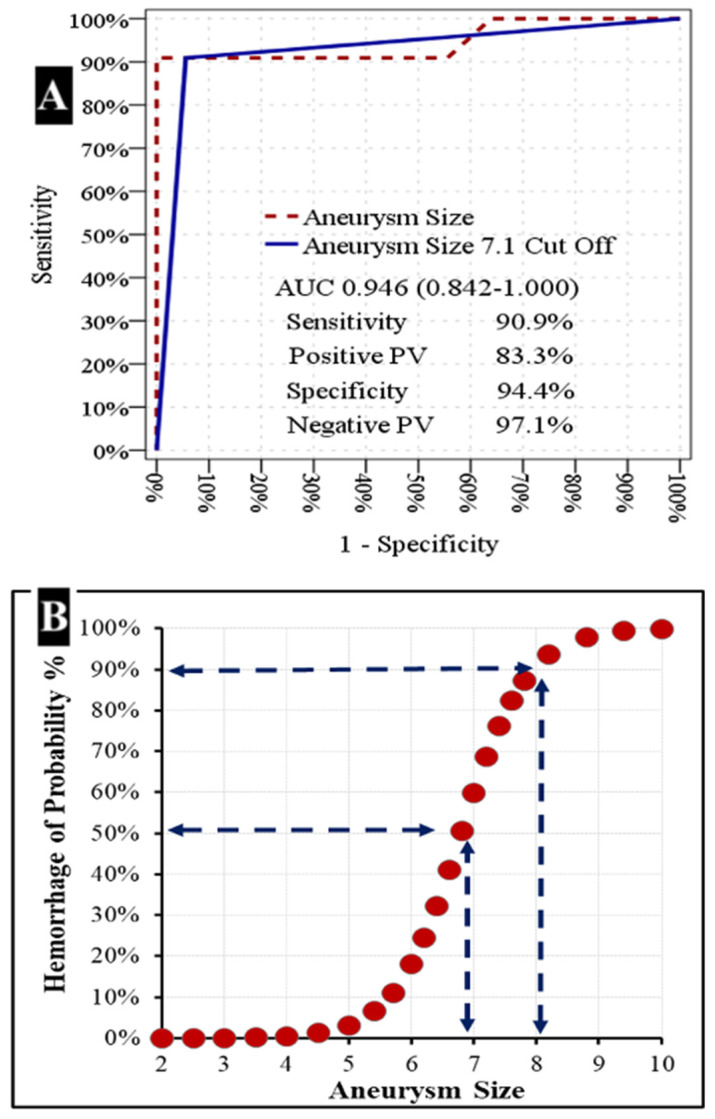
Relationship between aneurysm size and hemorrhage risk.

Aneurysm size demonstrated a significant ability to distinguish between patients with and without hemorrhage [area under the curve (AUC): 0.884 (0.827–0.941)]. Aneurysm size with a cutoff value of 7.1 also showed significant discriminative power between hemorrhagic and non-hemorrhagic patients [AUC: 0.824 (0.749–0.898)] (Table 3 and Figure 6). At the 7.1 cutoff value, the sensitivity was 90.9%, the positive predictive value was 83.3%, the specificity was 94.4%, and the negative predictive value was 97.1% in distinguishing between patients with and without hemorrhage (Table 3). These findings demonstrate that aneurysm size, particularly with a threshold of 7.1 mm, is a reliable predictor for the presence of hemorrhage.

(A) Receiver Operating Characteristic (ROC) Curve for Aneurysm Size. The ROC curve illustrates the diagnostic performance of aneurysm size in predicting hemorrhage. The solid blue line represents the performance of an aneurysm size cutoff of 7.1 mm, while the dashed maroon line represents aneurysm size as a continuous variable. The area under the curve (AUC) for aneurysm size is 0.946 (95% CI: 0.842–1.000). At the 7.1 mm cutoff, the sensitivity is 90.9%, positive predictive value (PV) is 83.3%, specificity is 94.4%, and negative PV is 97.1%.

(B) Probability of Hemorrhage as a Function of Aneurysm Size. This scatter plot demonstrates the increasing probability of hemorrhage with increasing aneurysm size. Dashed lines indicate that a hemorrhage probability of approximately 50% is observed at an aneurysm size of around 6.8 mm, and a probability of approximately 90% is observed at an aneurysm size of around 8.2 mm.

## 4. Discussion

**Figure 7:**
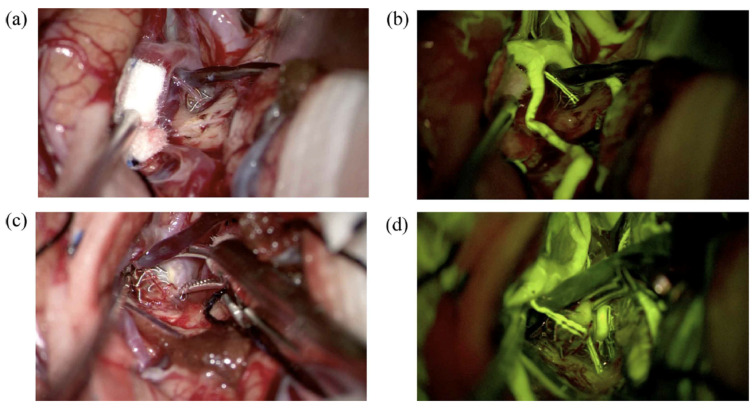
(**a**) A left MCA aneurysm encountered in a 45-year-old woman was coiled 3 years ago. A dog-ear-type remnant at the neck was observed on the follow-up, and she underwent surgery. The lenticulostriate artery in front of the aneurysm dome was seen under normal microscope light. (**b**) Same view under YELLOW 560 nm filter. (**c**) The aneurysm neck was clipped under normal microscope light. (**d**) A second Na-Fl videoangiography demonstrates that the lenticulostriate artery flow is patent following aneurysm clipping.

Intraoperative DSA has long been regarded as the gold standard in vascular neurosurgery for verifying aneurysm obliteration and assessing cerebral blood flow [10,11,12]. While it remains highly valuable in hybrid operating rooms, its routine use in standard surgical settings presents several challenges. The global adoption of hybrid operating rooms remains limited due to constraints such as the high cost of equipment and the relatively prolonged duration of the procedure [11,12]. The integration of intraoperative fluorescence-based videoangiography techniques has significantly enhanced the safety and efficacy of microsurgical aneurysm clipping. Among these, ICG and Na-Fl have emerged as valuable tools for the real-time visualization of vascular anatomy and confirmation of aneurysm obliteration [7,13,14]. ICG videoangiography, introduced by Raabe, has become widely adopted due to its high-resolution, near-infrared imaging capabilities [15]. Its ability to clearly delineate vessel patency and aneurysm neck occlusion intraoperatively has improved surgical outcomes and reduced the risk of incomplete clipping. However, the near-infrared light used for ICG imaging renders it invisible to the naked eye, requiring specialized camera systems and filters. Moreover, the signal penetration of ICG is limited, making deep or obscured vessel visualization suboptimal. On the other hand, sodium fluorescein, a fluorescent dye traditionally used in ophthalmology, has gained attention in neurosurgery due to its visibility under the yellow 560 nm filter integrated into modern surgical microscopes. Na-Fl videoangiography allows direct intraoperative assessment of vascular structures and clip placement without interrupting the flow of surgery. One of its notable advantages is its usability in standard optical fields without the need for infrared imaging systems [16,17]. Na-Fl facilitates real-time visualization of both superficial and perforating vessels and has been reported to be superior to ICG in detecting minor residual flow or branch compromise [18]. Unlike ICG, which operates in the near-infrared spectrum and is best suited for large vessel imaging, sodium fluorescein emits in the visible spectrum and provides continuous feedback during microsurgical dissection. Its use allows surgeons to visualize small perforators even during clip repositioning, without requiring interruptions or filter changes. This enhanced visualization helps guide clip placement with greater confidence and minimizes ischemic complications related to inadvertent occlusion of these critical vessels. In the present study, a patient who had previously undergone endovascular coiling for a left MCA aneurysm was operated on due to the detection of a remnant at the aneurysm neck on follow-up imaging. During the surgery, Na-Fl videoangiography clearly visualized the lenticulostriate artery intraoperatively, allowing for safe clipping of the aneurysm (Figure 7).

In the study published by Dashti and his colleagues, in which 239 aneurysms were surgically treated with the assistance of ICG videoangiography, occlusion of a major branch or perforating artery was identified in 15 aneurysms (6%), and 10 of these aneurysms (67%) were found to be located in the MCA [6]. In our case series, we did not encounter any instances of major branch or perforating artery occlusion, and we found that Na-Fl angiography was superior to ICG videoangiography in detecting perforating arteries.

**Figure 8:**
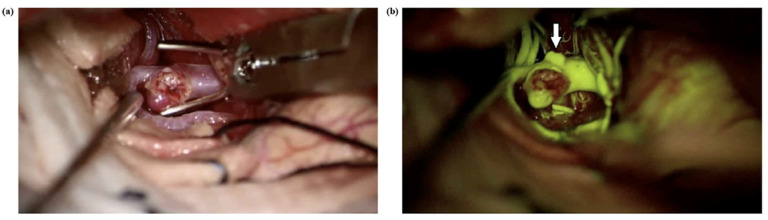
(**a**) A right MCA aneurysm in a 54-year-old man was shown before aneurysm clipping. (**b**) Na-Fl videoangiography under a YELLOW 560 nm filter shows an extra bleb on the aneurysm (arrow) dome, which was not visible under normal microscope light.

A primary limitation of Na-Fl videoangiography is its relatively prolonged intravascular persistence, which may hinder repeated assessments during multiple clip repositionings [19]. In the literature, Na-Fl has been reported to persist within the vessel wall for up to 20 min and even longer within the aneurysmal sac, and it has been emphasized that this may limit its effectiveness in evaluating aneurysmal dome filling following clipping [16]. In our study, consistent with the literature, the use of Na-Fl videoangiography both before and after clipping made it difficult to assess residual aneurysmal filling due to the persistence of the dye in the aneurysmal dome from the pre-clipping injection. However, ICG videoangiography does not share this limitation, as its rapid intravascular clearance permits repeated intravenous administrations within short intervals, thereby allowing reliable reassessment following clip repositioning [12]. Na-Fl videoangiography, when used with a dedicated 560 nm yellow filter in modern surgical microscopes, enables enhanced intraoperative visualization of vascular anatomy beyond what is possible with conventional white-light microscopy [7]. One of its key advantages is the ability to delineate the fine morphological features of the aneurysmal sac, such as small daughter sacs, lobulations, or bleb formations, which may not be apparent under standard illumination (Figure 8).

In the literature, there are publications emphasizing that ICG videoangiography provides poor image quality in deep operative fields and that there is a discordance between intraoperative videoangiography and postoperative DSA, particularly in AComA aneurysms [12,20,21]. Raabe and his colleagues conducted a comparative study between ICG videoangiography and intra- or postoperative DSA in the surgical management of 124 aneurysms across 114 patients at two neurosurgical centers [15]. Their analysis demonstrated a 90% concordance rate between ICG-VA and intraoperative DSA findings in a subset of 60 aneurysms. In our study, among 47 aneurysms treated with the assistance of intraoperative videoangiography, a small remnant was detected in two cases (4.26%) on postoperative DSA, which was also detected with intraoperative videoangiography. The technical quality of videoangiography was optimal for all of the anurysms and concordance of intraoperative videoangiography with postoperative DSA is 100%. Unlike previously reported findings in the literature, one of these aneurysms were located in the distal ACA and the other one was in MCA. A deliberate remnant was left in both aneurysms to preserve the patency of the distal branches and minimize the risk of compromising critical perforators or adjacent vessels.

The picket-fence clipping technique is particularly valuable in the surgical management of giant and complex aneurysms, where traditional neck clipping may be technically unfeasible due to factors such as calcification, atherosclerosis, wide-neck morphology, or involvement of multiple efferent branches. In our study, five (12.5%) giant MCA aneurysms were operated on using the picket-fence technique. Intraoperative ICG and Na-Fl angiographies showed that the parent arteries were patent, and no filling was observed in the aneurysms. The results were checked and confirmed with postoperative DSA. The combined use of both ICG and Na-Fl in the picket-fence technique ensures comprehensive intraoperative vascular assessment, minimizes the risk of ischemic complications, and increases the likelihood of complete aneurysm occlusion. These tools not only enhance surgical precision but also serve as real-time quality control measures, improving overall safety and efficacy in the treatment of challenging aneurysms.

## 5. Conclusions

Hypertension, smoking history, aneurysm size, and location were important predictors of aneurysm rupture. The use of intraoperative ICG and Na-Fl videoangiography provides real-time, high-resolution visualization of vascular anatomy during intracranial aneurysm surgery. These techniques are particularly valuable in complex cases such as giant aneurysms, where precise assessment of parent vessel patency and aneurysm exclusion is critical. In cases operated using the picket-fence clipping technique, both ICG and Na-Fl videoangiographies proved effective in confirming the complete occlusion of the aneurysm and the preservation of flow in adjacent vessels. The complementary use of these fluorescence-based modalities enhances surgical safety, supports intraoperative decision-making, and contributes to improved outcomes in the microsurgical treatment of intracranial aneurysms.

## Figures and Tables

**Table 1:** Patient demographics.

		Min–Max	Median	Mean ± sd/n-%
Age		30.0–70.0	54.0	51.8 ± 11.3
Gender	Female			n: 31 66.0%
	Male			n: 16 34.0%
Hypertension				n: 21 44.7%
Diabetus Mellitus				n: 12 25.5%
Family History of Subarachnoid Hemorrhage				n: 8 17.0%
Smoking History				n: 20 42.6%
Hemorrhage				n: 11 23.4%
Aneurysm Location	MCA			32 ± 68.1%
	AcomA			11 ± 23.4%
	PcomA			1 ± 2.1%
	ACA			3 ± 6.4%
Aneurysm Size		1.7–9.5	5.0	5.4 ± 1.8
Postoperative DSA Remnant Aneurysm				2 ± 4.3%

**Table 2:** Clinical characteristics and aneurysm features in hemorrhagic vs. non-hemorrhagic groups.

		Hemorrhage (−) (n: 36)		Hemorrhage (+) (n: 11)		*p*	
		Mean ± sd/n-%	Median	Mean ± sd/n-%	Median		
Age		52.9 ± 10.7	54.5	48.1 ± 13.0	49.0	0.218	^m^
Gender	Female	25 ± 69.4%		n: 6 54.5%		0.361	^X2^
	Male	11 ± 30.6%		n: 5 45.5%			
Hypertension	(−)	26 ± 72.2%		n: 0 0.0%		0.000	^X2^
	(+)	10 ± 27.8%		n: 11 100%			
Diabetus Mellitus	(−)	25 ± 69.4%		n: 10 90.9%		0.153	^X2^
	(+)	11 ± 30.6%		n: 1 9.1%			
Family History of Subarachnoid Hemorrhage	(−)	32 ± 88.9%		n: 7 63.6%		0.073	^X2^
	(+)	4 ± 11.1%		n: 4 36.4%			
Smoking History	(−)	26 ± 72.2%		n: 1 9.1%		0.000	^X2^
	(+)	10 ± 27.8%		n: 10 90.9%			
Aneurysm Location	MCA	26 ± 72.2%		n: 6 54.5%		0.271	^X2^
	AcomA	6 ± 16.7%		n: 5 45.5%		0.048	^X2^
	PcomA	1 ± 2.8%		n: 0 0.0%		1.000	^X2^
	ACA	3 ± 8.3%		n: 0 0.0%		1.000	^X2^
Aneurysm Size		4.6 ± 1.1	4.7	8.0 ± 1.4	8.5	0.000	^m^
Postoperative DSA Remnant Aneurysm	(−)	34 ± 94.4%		n: 11 100%		1.000	^X2^
	(+)	2 ± 5.6%		n: 0 0.0%			

^m^ Mann–Whitney U test. ^X2^ Chi-square test (Fischer test).

**Table 3:** Receiver operating characteristic (ROC) curve analysis and diagnostic performance metrics for aneurysm size in differentiating hemorrhagic from non-hemorrhagic patients.

		Area Under Curve		95% Confidence Interval			*p*
Aneurysm Size		0.884		0.827	-	0.941	0.000
Aneurysm Size 7.1 Cut Off		0.824		0.749	-	0.898	0.000
		Hemorrhage (−)	Hemorrhage (+)				%
Aneurysm Size	<7.1	34	1	Sensitivity			90.9%
	≥7.1	2	10	Positive PV			83.3%
				Specificity			94.4%
				Negative PV			97.1%
ROC Curve							

## Data Availability

Study data is unavailable due to privacy and ethical restrictions of our hospital.

## Abbreviations

Na-Fl: Sodium fluorescein, ICG: Indocyanine green, DSA: Digital subtraction angiography, SAH: Subarachnoid hemorrhage, CT: Computed tomography, AcomA: Anterior communicating artery, MCA: Middle cerebral artery, ACA: Anterior cerebral artery, PcomA: Posterior communicating artery, World Federation of Neurosurgical Societies: WFNS.
